# Characterizing Supportive Services Use by Caregiving Relationship Status

**DOI:** 10.1093/geroni/igab046.1529

**Published:** 2021-12-17

**Authors:** Jasmine Travers, Chanee Fabius

**Affiliations:** 1 New York University, New York, New York, United States; 2 Johns Hopkins Bloomberg School of Public Health, Baltimore, Maryland, United States

## Abstract

Informal caregivers of aging older adults experience a high degree of burden and strain. These emotional experiences often stem from stressful tasks associated with caregiving. Caregiving supportive services that target the provision of support for stressful tasks are instrumental in alleviating caregiving burden and strain. Research is limited on what types of caregiving supportive services caregivers are accessing by relationship status and their source of information. We sought to characterize caregiving supportive services use by caregiving relationship status. We analyzed cross-sectional data from the 2015 National Study of Caregiving limited to caregivers of older adults □65 years. Caregiver relationship status (i.e., spouse, child, other relative/non-relative) was the independent variable. Bivariate analyses were performed to examine the association with caregiver relationship status and 1) any use of supportive services, 2) type of supportive service used among users, and 3) source of information about supportive services. Our sample consisted of 1,871 informal caregivers, 30.7% reported using supportive services. By caregiver relationship status, children had the greatest use of supportive services compared to spouses and other relatives/non-relatives (33.3% vs. 22.5% vs. 22.1%, p=.02, respectively). Among users of services, there were no differences in type of services used. Spouses primarily received their information about services from a medical provider or social worker (73.8%, p=.004). Our findings highlight the need to ensure that other caregiving groups such as spouses have access to important supportive services such as financial support. Medical providers and/or social workers should be better leveraged and equipped to provide this information.

